# Data-Efficient Design of High-Entropy Oxygen Carriers for Chemical Looping Using Active Learning

**DOI:** 10.1021/acsmaterialsau.5c00230

**Published:** 2026-01-22

**Authors:** Joakim Brorsson, Henrik Klein Moberg, Joel Hildingsson, Jonatan Gastaldi, Tobias Mattisson, Anders Hellman

**Affiliations:** † Department of Physics, 11248 Chalmers University of Technology, SE-41296 Gothenburg, Sweden; ‡ Department of Space, Earth and Environment, 11248 Chalmers University of Technology, SE-41296 Gothenburg, Sweden

**Keywords:** materials discovery, active learning, oxygen carriers, chemical looping, high entropy oxides, machine learning potentials, first-principles

## Abstract

High-entropy materials, first demonstrated in metallic alloys and later extended to oxides and other systems, unlock a vast compositional space with properties suited for catalysis, energy, and structural materials. However, the high compositional complexity makes systematic exploration challenging, and only a small portion of the design space has been studied. To address this, we introduce an active learning strategy that integrates predictive modeling, uncertainty estimation, and iterative sampling to efficiently navigate embedded compositional material spaces. This approach continuously learns from previous evaluations, focusing subsequent searches on the most promising regions while reducing both time and data requirements. We demonstrate this methodology in the search for high-entropy oxygen carriers for chemical looping, where it rapidly accelerates discovery and identifies promising candidates more effectively than conventional trial-and-error or grid-search approaches. Importantly, this strategy is general and well-suited to exploring the vast space of multicomponent materials.

## Graphical abstract

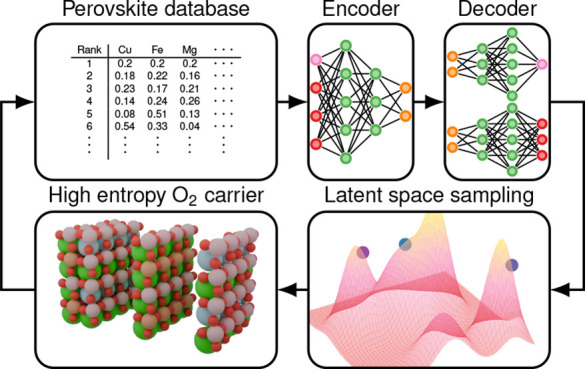

High entropy materials (HEMs) are, by definition, made up of five or more elements.^1,2^ The resulting compositional space combined with entropy-stabilized phases enables a shift from “using the material you have” to “engineering the material you need”.^3^ By virtue of simple combinatorics, the compositional space of such complex materials can be enormous,^4^ making traditional approaches to materials discovery ineffective, and calls for more advanced strategies to accelerate the process. This is especially important for materials that enable next-generation energy conversion technologies, which must be developed rapidly to address environmental and climate challenges.^5^ Recently, various machine learning (ML) methods have been utilized, allowing, e.g., the discovery of up to several hundred thousand stable compounds by scanning millions or billions of material candidates.^6–8^ While these efforts mark major progress, they demand substantial computational resources and training data, and even such large-scale searches still probe only a minute fraction of the enormous compositional space of HEMs. In response to this challenge, Rao et al.^9^ introduced an active learning (AL) strategy, which requires only sparse data for training, to explore high entropy Invar alloys, revealing several candidates exhibiting both thermal stability and low thermal expansion coefficients. Drawing inspiration from this work, the present study aims to demonstrate how AL can be generalized to accelerate the search for any type of HEM. Central to this generalization is the use of adaptable material property functions, e.g., stability, phase transitions between reduced and oxidized states, configurational entropy, or elemental abundance, which enable steering the AL cycle toward the most relevant regions of the design space.

As a proof of concept, we apply the approach to identify next-generation high entropy oxides (HEOs) to be used in chemical looping (CL), a process for efficient fuel conversion which can be applied to combustion, gasification, and reforming. CL has been suggested as a breakthrough technology for achieving carbon capture and storage at low cost.^10^ The core idea is to utilize a solid oxygen carrier (OC), typically a metal oxide, to separate the conversion process into two distinct chambers: one for air and one for fuel^11–13^ (see Figure S1). As it mechanically circulates between these reactors, the OC is oxidized in the air reactor and reduced in the fuel reactor. The OC particles must have redox energetics compatible with the process, enabling oxygen uptake in the air reactor and release in the fuel reactor. Further, the OCs must have sufficient oxygen transfer capacities (OTCs) to convert the fuel to the desired products. The initial focus was on monometallic oxides based on transition metals Ni, Fe, Cu, Mn, and Co.^10^ To overcome restrictions with respect to stability and reactivity, in the past decade, a number of higher-order systems, including cubic HEOs and ABO_3_-type perovskites, have been studied by several different groups, leading to the identification of some promising candidates.^14,15^ Still, it should be emphasized that CL is still at a relatively low technical readiness level, with most experiments performed in small pilot units, with OC stability and reactivity being major barriers for up-scaling. So far, most OC development has been based on simple thermodynamics and “trial-and-error” approaches, strategies that become unmanageable as the compositional space increases. Although there have been some high-throughput studies for the discovery of new OC materials,^13,16,17^ no studies using AL approaches have been published. Hence, it is of central importance to find efficient methods to accelerate the development of higher-order OCs.

The focus in this study is ABO_3_ perovskites, a class of materials that have been shown to be viable as OCs, and which can also be doped in order to achieve high-entropy configurations. Here, A represents a larger and B a smaller cation that are coordinated by 6 and 12 oxygen atoms, respectively. The stable crystal structure is also advantageous in CL applications since it allows oxygen transfer to occur via the formation of defects rather than phase transformations, in contrast to simple monometallic systems.^18^ Even so, the introduction of additional elements, on either or both sublattices, causes an exponential increase in possible configurations (Figure 1a), compromising the tractability after just a few additions. This is accompanied by a steady rise in the maximum configurational entropy, underscoring the high-entropy character attained via cation mixing on multiple sublattices (Figure 1a). A comparison of literature-reported estimated and experimental OTC values shows good agreement across a range of compositional complexities, as demonstrated by comparing oxides and perovskites with one to four distinct cations (see Figure 1b and Table S1). Notably, multicomponent perovskite OCs (e.g., quaternary oxides) show similar or better performance than simpler single- or binary-metal oxides. Hence, there are compelling reasons to construct efficient AL strategies to identify high-entropy OCs. Still, caution should be emphasized when comparing computational predictions with experimental data, as there exist many sources of error in both cases. In the present study, it has been necessary to balance speed and accuracy in order to test and optimize AL framework, since this requires sizable samples to be produced over multiple cycles without expending unreasonable time and computational resources. Consequently, a conservative limit has been used for the nonstoichiometry, i.e., δ ≤ 0.5 in ABO_3_, which not only agrees well with measurements for CaMnO_3_ ^19^ but also, more importantly, ensures compliance with the study of Wang et al.^16^ While δ values as high as ∼2.0 have been reported for some chemistries, evidence that the perovskite structure is retained after the reduction is seldom provided;^20–24^ in fact this has been proven not to be the case for, e.g., MgMnO_3−δ_ and CaMn_0.9_Mg_0.1_O_3−δ_.^25,26^ As the goal of the present study is to identify the best candidates via ranking, systematic errors, such as the assumption of a too low maximum for δ, will have a limited impact on the final results.

**Figure 1:**
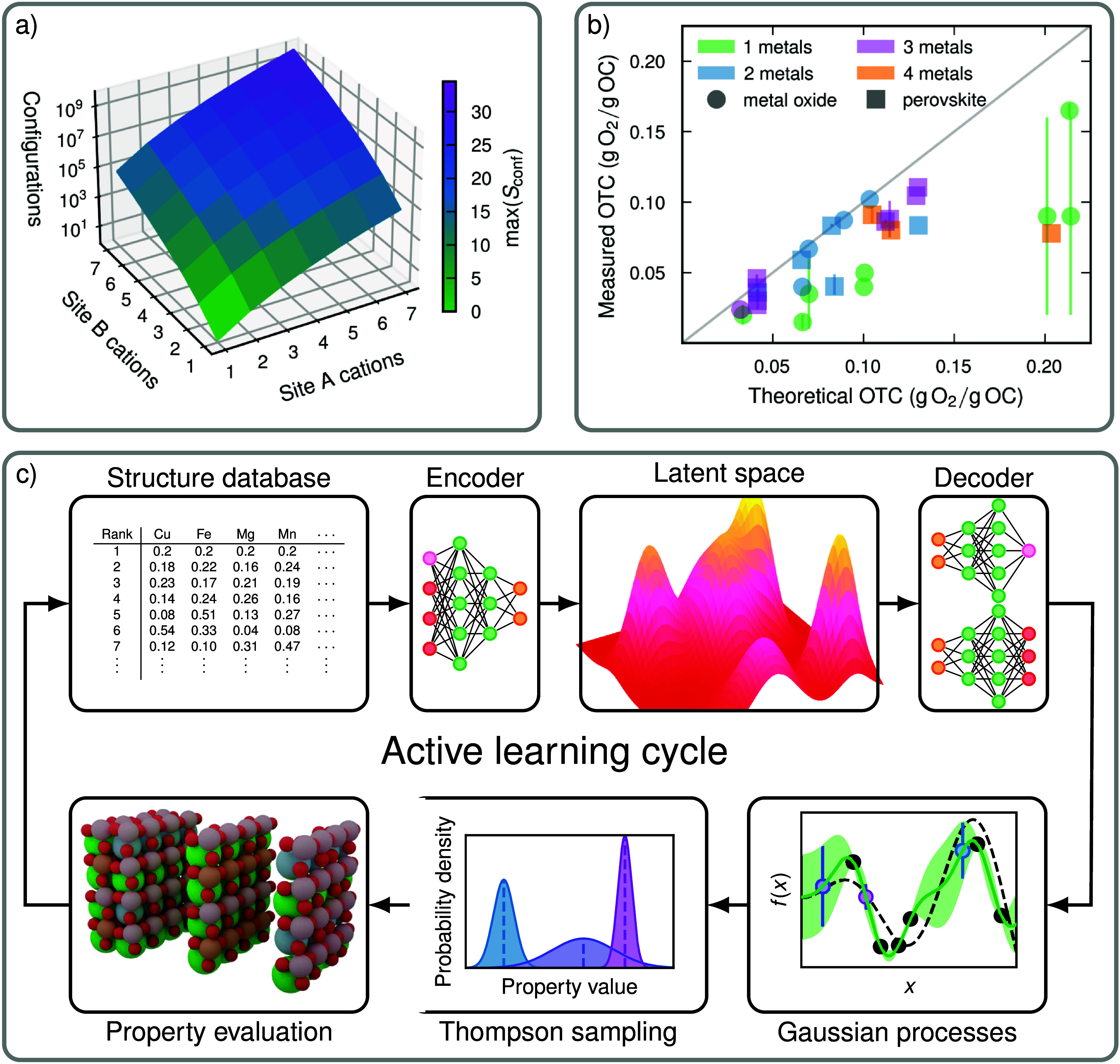
(a) Number of possible configurations together with the maximum configurational entropy for an 80-atom ABO_3_ perovskite supercell plotted against the number of different cations that share the A and B sites. (b) Measured versus theoretical OTCs reported for various oxides (circles) and perovskites (squares) featuring one (green), two (blue), three (purple), or four (orange) distinct metallic elements (see Table S1).^20–26,31–52^ (c) Schematic illustration of the AL cycle, in which the first step is to encode a database of compositions and target properties into a continuous latent space via a WAE-GMM model. Then Thompson sampling is used to select candidates, based on estimated property values and uncertainties obtained from GP. Following validation, with CHGNet, the results, which include updated property values, are fed back into the database, enabling iterative learning and refinement.

The AL model (see Figure 1c) consists of a Wasserstein autoencoder (WAE). Specifically, its task is to learn the latent representation of the data, which is modeled using a Gaussian mixture model (GMM) to ensure that the corresponding space is continuous, tractable, and chemically informative (see Figure S2). At this stage, sampling is performed within the latter to identify points that are proximal to the original data manifold and, thus, likely to represent chemically valid compositions. Gaussian processes (GP) is subsequently applied to assess uncertainty and facilitate the selection of promising compositions via Thompson sampling,^27^ thereby balancing exploration and exploitation. Candidates are ranked according to their predicted target properties, and subsequently evaluated with respect to their thermodynamic properties (see Figure S3) using CHGNet,^28^ a state-of-the-art machine learning interatomic potential (MLIP) trained on 1.6 million entries from the Materials Project.^29,30^ Although first-principles or experimental validation could, in principle, be employed, CHGNet was chosen to enable faster iteration and screening. Finally, the evaluated candidates—with updated target properties—are incorporated into the database. Through iterative cycles of model training, probabilistic candidate generation, and property evaluation, this AL strategy rapidly pinpoints promising high entropy oxygen carriers (HEOCs) from a myriad of possible ABO_3_ configurations.

To highlight the AL strategy and its data efficiency, we first focus on the prediction of HEOCs based on ABO_3_ perovskites for reforming via CL for production of H_2_ and CO_2_. Here, a limited training data set from the study by Wang et al.^16^ was utilized. In their study, the design space of perovskite oxides was systematically expanded by taking SrFeO_3_ as a parent structure and exploring extensive A- and B-site substitutions through high-throughput density functional theory (DFT) calculations. This substitution strategy yielded a diverse data set of approximately 2400 compositions, several of which exhibited superior redox behavior compared to the baseline SrFeO_3_.

We randomly select only 10%—a limited collection of ∼240 candidates—as our initial database. Additionally, the entries in this database are updated with the probability of the candidate being suitable for dry reforming using CL (see Methods section), which will be the target property optimized during the cycles. Candidates were filtered for charge neutrality and Goldschmidt tolerance in each cycle. After five parallel runs of 30 cycles each, approximately 200 new OCs had been identified, which demonstrated the strong data efficiency. Compared to a purely random or unguided strategy (see Figure 2a), the histogram of discovered materials from the WAE-GMM model with Thompson sampling is skewed toward the upper end of the probability range, indicating that it identifies significantly more high-probability OCs (see Figure 2b). This underscores the efficacy of AL in navigating the compositional space. Further analysis of the candidates selected by this approach shows that the top-ranking compositions (those with the best predicted dry reforming capability) tend to exhibit a high compositional complexity, often containing five or more different cations. This is evident in the all-candidate averages per cycle, which show a steady increase in configurational entropy, decreases in mean metal abundance, and higher probability with each iteration (Figure 2c–e). In other words, the algorithm progressively shifts toward more compositionally diverse (but less common) regions of the search space in the pursuit of higher predicted performance materials.

**Figure 2:**
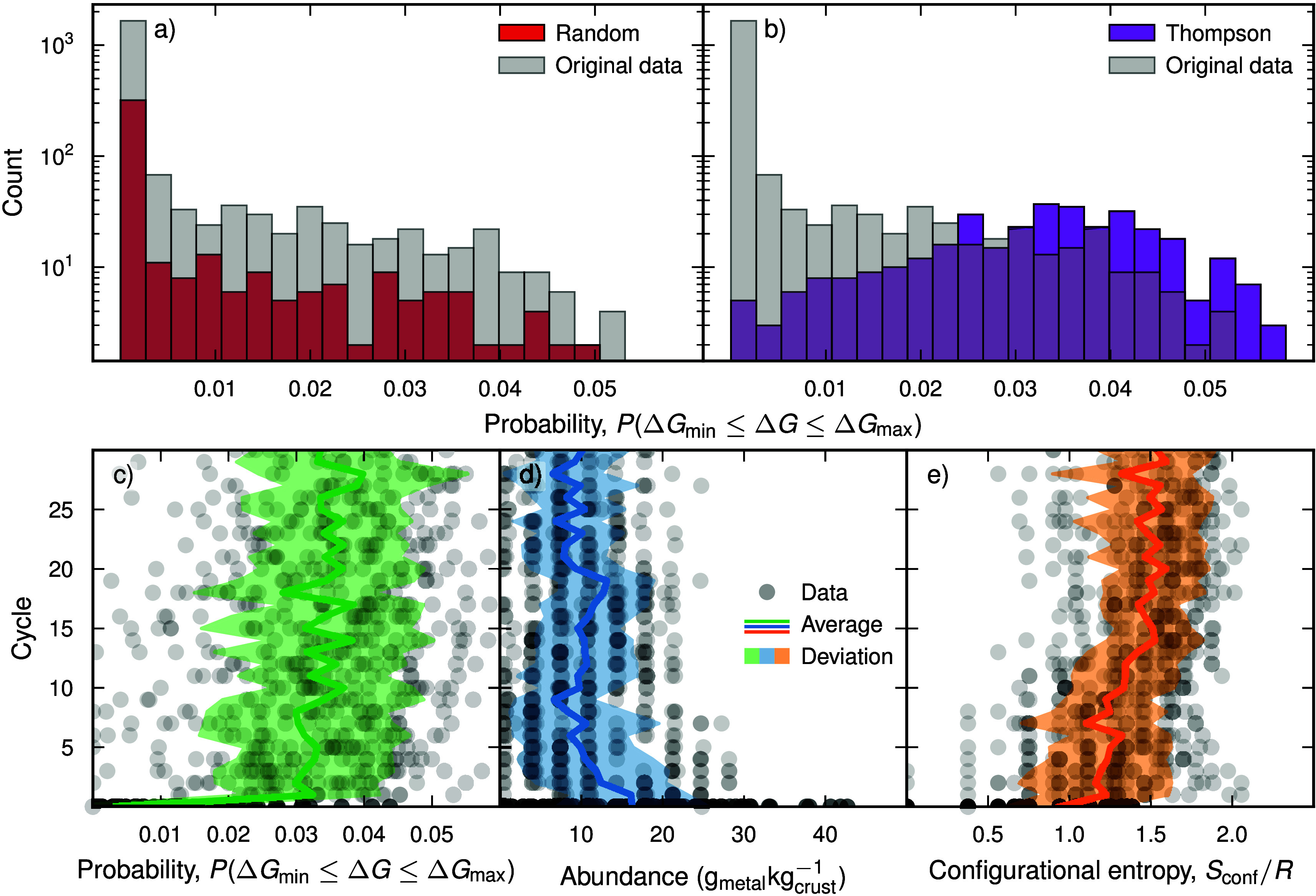
Diagrams with counts of the number of unique candidates with a given value on the target property, i.e., the probability of being suitable as an OC for dry reforming using CL, for the new materials identified when using random (a) and Thompson (b) sampling. Furthermore, the probability (c), average abundance of the metallic elements in the earth’s crust (d), and configurational entropy, i.e., *S*_conf_/*R*, (e) are displayed for the original (gray circles) and added (purple circles) data when using the latter method. Here, the averages (solid line) and standard deviations (filled curve) are also indicated.

The results in Figure 2 clearly demonstrate the advantages of AL strategies over traditional methods, i.e., “trial-and-error” or grid search. Even so, the probability-based measure that we used as a target value, while useful for guiding experiments, is difficult to compare with measurements. To demonstrate that the methodology is equally effective for directly measurable properties, we also addressed the problem of identifying HEOCs with a maximum of OTC under chemical looping combustion (CLC) conditions. In this case, it is necessary to leverage two additional properties: the configurational entropy and the Bartel tolerance factor (τ). The former should, more precisely, not exceed 1.5R in order to satisfy the HEM criteria, while the latter, which predicts the stability and synthesizability of ABO_3_ perovskite structures, was strictly required to be lower than 4.18—candidates with a higher value were filtered out.

In order to achieve more accurate OTC estimates, the representative special quasi-random structures (SQSs) were based on larger (80-atom) supercells and covered multiple crystal symmetries, i.e., orthorhombic, cubic, and brownmillerite. In addition, a broader training data set was constructed, to obtain a better starting point for the model, by generating 1815 random ABO_3_ configurations, of which 1066 remained after those with low configurational entropy and high Bartel tolerance had been filtered out. The same AL framework was employed, although the design space was expanded by many orders of magnitude owing to the larger supercell. To enable a broader search, five independent runs of 10 cycles each were carried out using identical training sets but different random seeds. The resulting candidates were merged into a single data set, which was subsequently advanced through 40 additional cycles (see Figure 3).

**Figure 3:**
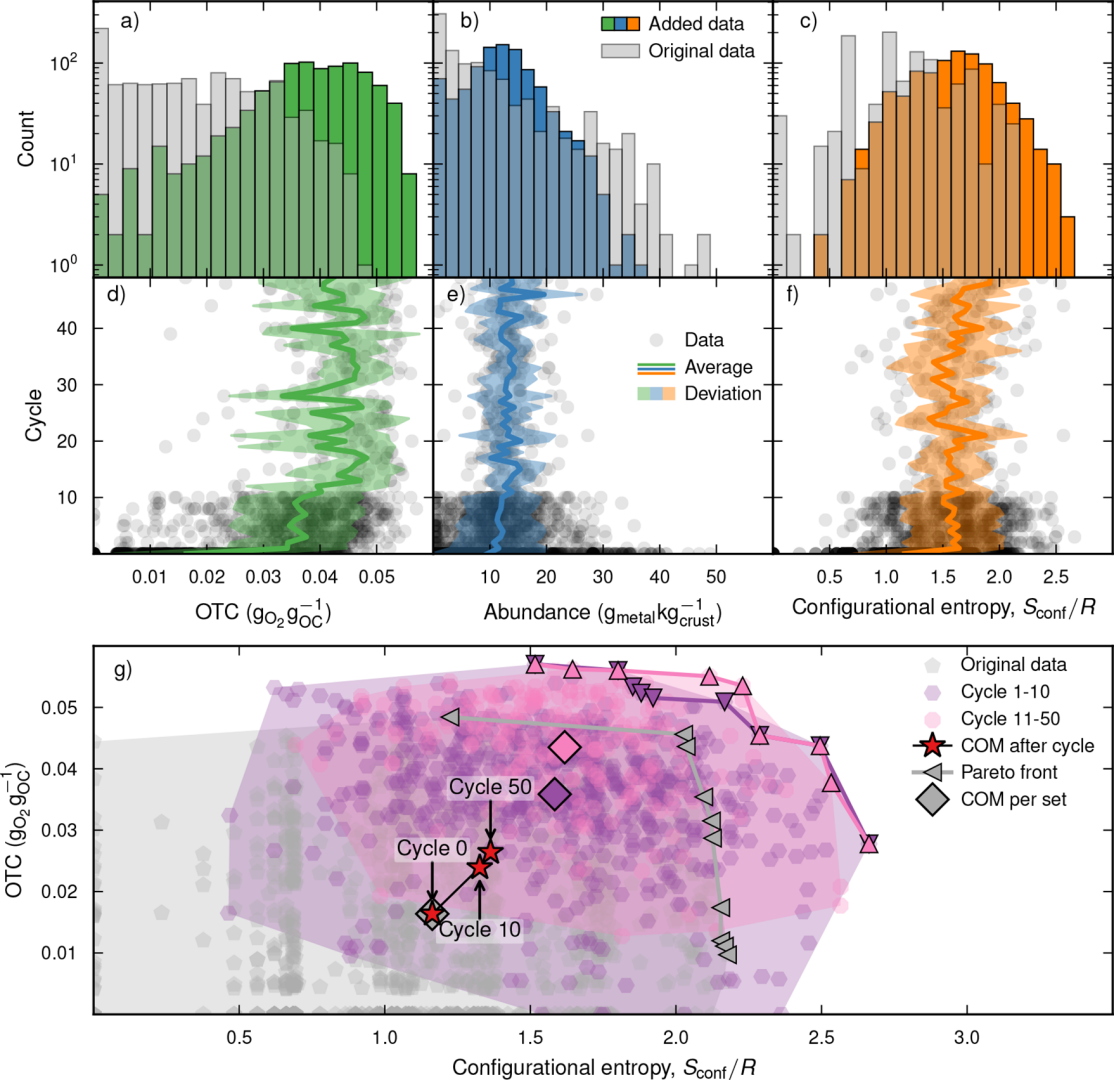
Diagrams with the computationally verified OTC (a,d); average abundance of the metallic elements in the earth’s crust (b,e); and configurational entropy, *S*_conf_/*R*, (c,f) versus counts of the number of unique candidates (a–c) and those added in each cycle (d–f) as well as a Pareto plot (g). Specifically, this includes data from the original training set (gray circles) together with cycles 1–10 (purple circles) and 11–50 (pink circles). In the second row, averages (solid line) and standard deviations (filled curve) are also shown. Additionally, the Pareto fronts (triangles) as well as the center-of-mass for the individual sets (diamonds) and the accumulated data up to a certain cycle (red stars) are displayed in the last panel.

It is important to note that the AL algorithm was optimized solely with respect to the OTC while still fulfilling the criteria for the different CL processes. Nevertheless, the training procedure led to the selection of structures with both lighter elements and increased configurational entropy compared to the initial data set (see Figure 3a–f). This demonstrates that the WAE-GMM model combined with Thompson sampling is highly effective at identifying promising candidates. Notably, top-performing materials such as Ca_13_CoMgMn_9_Ti_5_Y_3_O_48_, Ca_11_Mg_3_Mn_8_Ti_5_Y_5_O_48_, and Ca_11_FeMg_3_Mn_8_Ti_4_Y_5_O_48_ achieved OTC scores of 0.0570 g_O_2__g_OC_^–1^, 0.0562 g_O_2__g_OC_^–1^, and 0.0560g_O_2__g_OC_^–1^, respectively, and interestingly, all include Ca and Mn—elements well-known for their strong oxygen-carrying capabilities. The fact that the Pareto front successively expands with respect to both the OTC, as obtained from the first-principles calculations, and configurational entropy provides further evidence of the strength of AL (see Figure 3g). Indeed, the majority of the added points, after 10 cycles, have a configurational entropy *S*_conf_ ≥ 1.5*R* and an OTC of at least 0.03g_O_2__g_OC_^–1^. Moreover, this trend grows stronger with the number of cycles.

Taken together, our results illustrate that AL provides an efficient and robust framework for discovering new materials, especially in systems in which the configurational space is vast. We have not only discovered many promising OCs for dry reforming and CLC but also shown how the generative model improves steadily over the training cycles. Thanks to its inherent flexibility, this approach can be readily extended to other material classes as well as applications and adapted by, e.g., replacing or combining ab initio calculations with experiments. We therefore envision that AL strategies that integrate WAE-GMM with Thompson sampling will become increasingly important in both applied and fundamental materials science, thereby advancing the design of multicomponent oxides for sustainable energy technologies.

## Method

In the sections that follow, we provide a condensed overview of the methodology employed in this study; a more detailed description can be found in reference 53.

### Active Learning

We use AL to refine a surrogate model, which predicts material properties and estimates the associated uncertainty, in a way that balances exploration and exploitation (for more details, see Note S1). An acquisition function is applied for querying in each round, whereafter the surrogate is updated. Here, Thompson sampling is utilized, which is designed to sample the posterior of the model based on the identification of promising or informative points because of its simplicity and effectiveness.^27,54^ To generate candidates, we have, more precisely, chosen to couple a WAE^55^ with a GMM. The rationale for this choice is to take advantage of the former architecture and the adaptable encoding offered by the latter. In this way, we obtain a flexible yet structured latent representation with the capacity to capture the intricacies of compositional relationships. The trained WAE can thus be conditioned to sample compositions, in a controlled manner, that are not only chemically reasonable but also likely to have desirable target properties.

### Structure Generation and Filtering

Similarly to Wang et al.,^16^ the training data used for testing the AL methodology for CL dry reforming consisted of 2401 cubic 40 atom Sr_1–*x*_A_*x*_Fe_1–*y*_B_*y*_O_3−δ_ supercells, with Sr and Fe partially substituted by A = Ca, K, Y, Ba, La, Sm and B = Co, Cu, Mn, Mg, Ni, Ti, respectively. In particular, this meant that *x* and *y* ranged from 0 to 1 in steps of 0.0125. The same applies to the oxygen nonstoichiometry δ, except that the upper limit was 0.5 instead. In addition, the brownmillerite structure, Sr_2–2*x*_A_2*x*_Fe_2–2*y*_B_2*y*_O_5_, was included for temperatures below 800 °C, to account for a possible decomposition when δ → 0.5. When attempting to find HEOCs for CLC, however, 1815 random ABO_3_ 80 atom supercells were generated to form the original training data. This meant that the step size in the oxygen nonstoichiometry was reduced to 0.0625. In addition, orthorhombic as well as cubic symmetries were considered together with the brownmillerite structure. Hence, the number of distinct SQSs evaluated for each composition was increased from 7 to 19, compared with the aforementioned verification study. Note that the ground state structures of SrFeO_3_ and Sr_2_Fe_2_BO_5_ in the Materials Project^29,30^ were consistently used as starting points regardless of the actual composition.

In agreement with Wang et al.,^16^ unstable compositions were excluded according to charge balance (Δ*q*_*e*_) together with a tolerance factor. Specifically, all initial training structures, as well as the proposed CLC candidates during the AL cycles, were evaluated based on the Bartel tolerance

$$\tau =\frac{{\mathrm{r̅}}_{X}}{{\mathrm{r̅}}_{B}}-{\mathrm{n̅}}_{A}\left({\mathrm{n̅}}_{A}-\frac{{\mathrm{r̅}}_{A}/{\mathrm{r̅}}_{B}}{\ln\left({\mathrm{r̅}}_{A}/{\mathrm{r̅}}_{B}\right)}\right)$$ 1

where *r̅*_*i*_ and *n̅*_*i*_ are the average radius and oxidation state on site *i* ∈ {A, B, X} in ABX_3_; presently X is occupied by oxygen atoms and vacancies.^56^ When considering CL dry reforming, enforcing Δ*q*_*e*_ = 0 and τ ≤ 4.3 narrowed the size of the predefined data set from 2401 to 2069. Also note that the Goldschmidt tolerance factor

$$\tau =\frac{{\mathrm{r̅}}_{A}+{\mathrm{r̅}}_{X}}{\sqrt{2}\left({\mathrm{r̅}}_{B}+{\mathrm{r̅}}_{X}\right)}$$ 2

was used to assess the stability of the added compositions in this case. For CLC, meanwhile, both charge neutrality and a Bartel tolerance of τ ≤ 4.18 were consistently required, leaving only 1066 of the 1815 original training structures.

### Property Estimates

The universal CHGNet MLIPs^28^ was utilized for the force and energy calculations. In addition, the ASE implementation^57^ of the Broyden–Fletcher–Goldfarb–Shanno (BFGS) algorithm was used for all structural relaxations, thereby ensuring that the interatomic forces did not exceed 0.001 eV Å^–1^. The lattice heat capacity, on the other hand, was estimated based on the harmonic approximations using the phonopy^58^ package. More precisely, this involved training force constants using a reciprocal grid with a density of 1000000 atom^–1^ from data obtained by imposing 0.02 Å displacements on specific atoms in supercells with spatial dimensions of at least 12.5 Å.

To assess the probability that a given candidate was suitable for dry reforming, the vacancy formation energies, $\Delta G_{\delta_{1}\to \delta_{2}}=G_{f}^{\delta_{2}}+\frac{\Delta \delta }{2}\mu_{O_{2}}-G_{f}^{\delta_{1}}$ associated with the reactions

$${\mathrm{Sr}}_{1-x}A_{x}{\mathrm{Fe}}_{1-y}B_{y}O_{3-\delta_{1}}⇋{\mathrm{Sr}}_{1-x}A_{x}{\mathrm{Fe}}_{1-y}B_{y}O_{3-\delta_{2}}+\frac{\delta_{2}-\delta_{1}}{2}O_{2}$$ 3

were compared with energy intervals defined by Wang et al.^16^ When calculating Gibbs free energy (*G* ^δ_*i*_^), the volume contribution *PV*_δ_*i*__^0 *K*^ as well as the discrepancy between the specific heat capacity at constant volume and pressure (*C*_*V*_^δ_*i*_^ ≈ *C*_*P*_^δ_*i*_^) were neglected. Consequently

$$G^{\delta_{i}}\left(T\right)\approx U^{\delta_{i}}\left(0\;K\right)+\int_{0}^{T}C_{V}^{\delta_{i}}\left(T^{\prime }\right)dT^{\prime }+S^{\delta_{i}}\left(0\;K\right)+\int_{0}^{T}\frac{C_{V}^{\delta_{i}}\left(T^{\prime }\right)}{T^{\prime }}dT^{\prime }$$ 4

where *U* ^δ_*i*_^(0 K) and *S* ^δ_*i*_^(0 K) are the internal energy and entropy at 0 K, respectively.

The formation energies were converted into measures of the OC capability by treating the formation energies as mean values (μ) of randomly distributed variables $X∼N\left(\mu ,\sigma^{2}\right)$ with a standard deviation of σ = 0.4 eV. The probability that any of the transitions with δ_2_ – δ_1_ ≡ Δδ = 0.125 were located within the interval *a* < Δ*G*_δ_1_→δ_2__ ≤ *b* is, thus, given by

$$P\left(a<X\leq b\right)=F_{X}\left(b\right)-F_{X}\left(a\right)=\frac{1}{\sigma \sqrt{2\pi }}\int_{-\infty }^{x}\exp\left(-\frac{{\left(t-\mu \right)}^{2}}{2\sigma^{2}}\right)dt{|}_{x=a}^{b}$$ 5

where *F*_*X*_(*x*) = *F*(*x*; μ, σ) is the corresponding cumulative distribution function, while (*a*, *b*) = (1.93 eV, 2.74 eV) at 800 °C and (*a*, *b*) = (1.83 eV, 2.91 eV) at 950 °C. Note that the interpolated formation energies at δ ∈ {0.0625, 0.1875, 0.3125, 0.4375} were also used in this evaluation.

As a measure of CLC capacity, the OTC was calculated from the difference in the nonstoichiometry (Δδ = δ_red_ – δ_ox_) under reducing (*T* = 1050 °C, *p*_O_2__ = 10^–14^ atm) and oxidizing (*T* = 950 °C, *p*_O_2__ = 0.2 atm) conditions. To be specific, the OTC value is 0.5Δδ*M*_O_2__/*M*_ABO_3__ where *M*_*i*_ is the molar mass of compound *i*.

To obtain a measure of candidate practical viability, the average metal abundance was deemed a better alternative than the material cost due to the inherent volatility in the market prices for individual elements. Specifically, the former was calculated as

$$\mathrm{X̅}=\frac{\underset{\mathrm{Me}}{\sum }n_{\mathrm{Me}}X_{\mathrm{Me}}}{\underset{\mathrm{Me}}{\sum }n_{\mathrm{Me}}}$$ 6

where *n*_Me_ is the number of atoms of type Me while *X*_Me_ is the corresponding abundance in the earth’s crust.^59^

## Supplementary Material



## Data Availability

Additional information, including a more elaborate account of the methodology, can be found in ref 53.

## Notes

Supporting material that illustrates key aspects of the study, including an overview of the chemical looping combustion process; a schematic of the WAE-based active learning pipeline, as well as a description thereof; and a detailed diagram outlining the procedure used to evaluate the properties of candidate materials (PDF)
