# Supplementary material for: Data-Efficient Design of High-Entropy Oxygen Carriers for Chemical Looping Using Active Learning
Source: ACS Mater Au. 2026 Jan 22;6(2):319–26. doi: 10.1021/acsmaterialsau.5c00230 (PMC12983108; doi:10.1021/acsmaterialsau.5c00230)
Supplement: Supplementary file 1 [file mg5c00230_si_001.pdf]

Supporting Information

Data-Efficient Design of High-Entropy Oxygen Carriers for Chemical Looping Using Active Learning

Joakim Brorsson[1], Henrik Klein Moberg[1], Joel Hildingsson[1], Jonatan Gastaldi[2], Tobias Mattisson[2], and Anders Hellman[1]

[1]Chalmers University of Technology, Department of Physics, SE-412 96 Gothenburg, Sweden

[2]Chalmers University of Technology, Department of Space, Earth and Environment, SE-412 96 Gothenburg, Sweden

Contents

Supporting Figures **2**
- S1. Illustration of chemical looping combustion . . . 2
- S2. WAE employed for the active-learning pipeline . . . 3
- S3. Workflow for property evaluation . . . 4

Supporting Tables **5**
- S1. Experimental oxygen transfer capacities . . . 5

Supporting Notes **6**
- S1. Active learning workflow . . . 6

Supporting References **8**

Supporting Figures

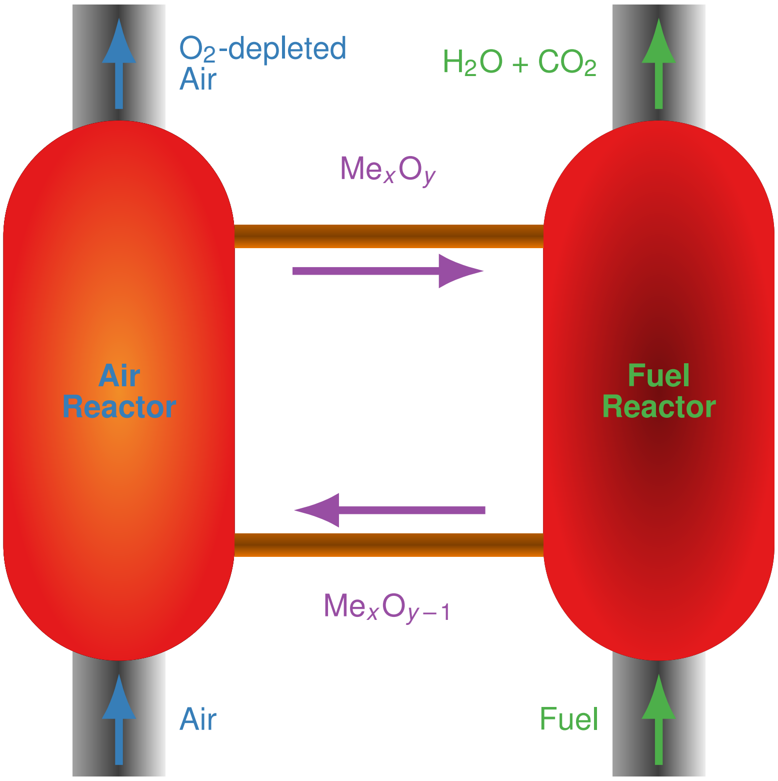

Figure S1: Illustration of chemical looping combustion. Schematic illustration of the chemical looping combustion (CLC) process. The core component is the oxygen carrier (OC), often in the form of a metal oxide (Me_xO_y), which is circulated between two chambers. Within one cycle, the material is first reduced by the fuel before being transported to the second reactor where it is reoxidesed thanks to the inlfux of air. As a result, one obtains a main output stream consisting only of water vapour and CO_2, which can, in turn, be easily separated via, e.g., condensation.

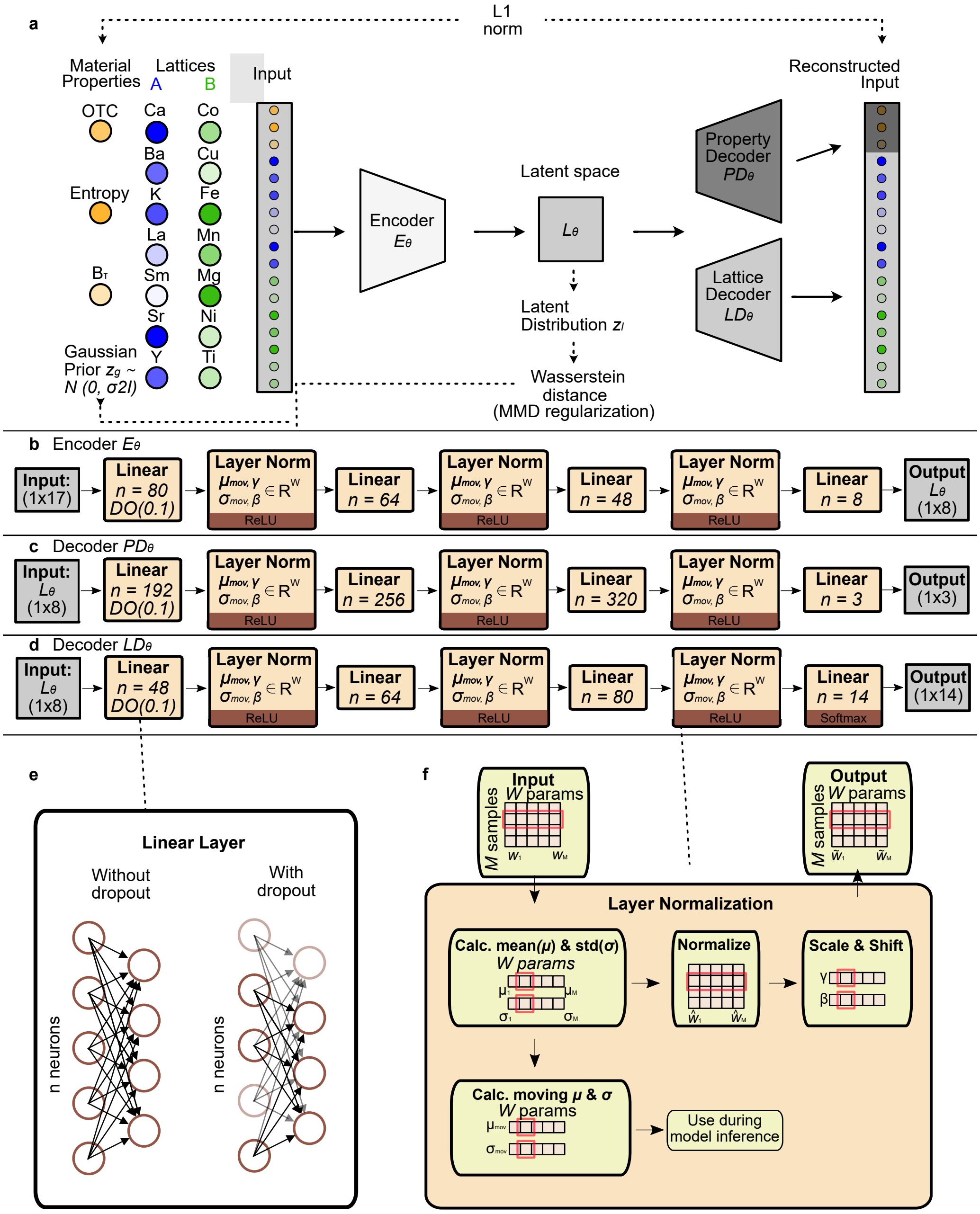

Figure S2: WAE employed for the active-learning pipeline. Overview of the Wasserstein auto-encoder (WAE) (1) active learning pipeline where the first part (a) shows the overall data flow. Specifically, the starting point is a 17-dimensional input vector composed of three material-property scalars, i.e., the oxygen transfer capacity (OTC), configurational entropy, and Bartel tolerance (B_τ) (2), and two 7-component soft-composition vectors for the A- and B-cation sublattice. This vector is passed through the encoder E_θ that maps it to an 8-dimensional latent variable L_θ. The latter is, in turn, compared to samples from a Gaussian prior by an maximum mean discrepancy (MMD) (1) term, and fed into two parallel decoders: one for properties (PD_θ) and another for lattice compositions (LD_θ). The training objective is the sum of an L_1 reconstruction loss (applied to the full 17-component output) and the MMD regularisation. Panel (b) gives the entire encoder architecture, encompassing fully-connected layers of $80 \to 64 \to 48 \to 8$ neurons. Each is followed by layer normalization (LN) (3) as well as rectified linear unit (ReLU) activation and, in the case of the first layer, a 10 % dropout. The property and lattice decoders, meanwhile, are depicted in (c) and (d) respectively. Although both are designed to mirror the encoder blocks, the former features a 4× scaled hidden width yielding latent $8 \to 192 \to 256 \to 320 \to 3$ neurons compared to $8 \to 48 \to 64 \to 80 \to 14$ for the latter. In the lattice encoder, the final layer is, moreover, split and normalised with a separate softmax for each sublattice to yield physically valid composition fractions. A conceptual sketch of a linear layer is provided in (e), to highlight the effect of dropout on synaptic connectivity. The last panel (f) illustrates the LN (3), including the batch-wise mean and variance estimation during training as well as the learned scale-and-shift parameters (γ, β) used at inference.

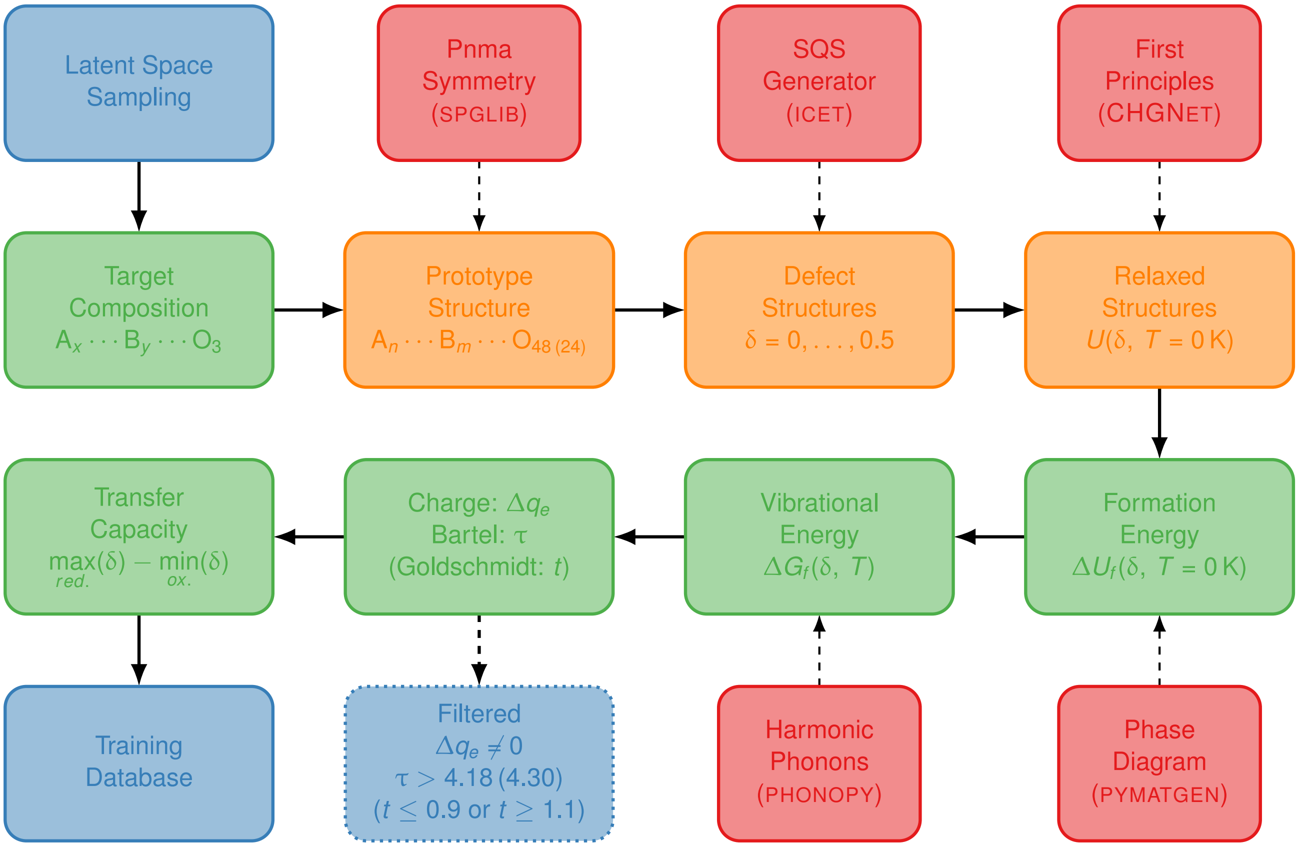

Figure S3: Workflow for property evaluation. Schematic diagram showing the procedure for evaluating the properties of candidate materials. Specifically, the first step is to obtain the target composition by, e.g., sampling the latent space, which is subsequently converted into a prototype structure by using an appropriate supercell as a template. Next, special quasi-random structures (SQSs) representing the various symmetries and defect concentrations of interest are generated and before being relaxed using a suitable method, here in the form of the CHGNET machine learning interatomic potential (MLIP) (4). By combining the results with data found in the Materials Project database, using PYMATGEN (5), a phase diagram can be constructed from which the formation energy of the material, at 0 K, can be extracted. Thereafter, harmonic phonon calculations are performed, with the help of PHONOPY (6), to determine the vibrational contribution to the free energy. This is followed by a filtering processes, which is, in this case, based on the total charge together with either the Bartel or Goldshmidt tolerance factor. Finally, the OTC is estimated for the remaining candidates, by comparing energies of formation for the different SQSs under oxidizing and reducing conditions, before they are added to the training database.

Supporting Tables

Formula	OTC	Reference
Fe_2O_3/Fe_3O_4	0.013 – 0.027	(7–10)
Fe_2O_3/FeO	0.04	(11, 12)
Mn_3O_4/MnO	0.01 – 0.06	(8, 13, 14)
CuO/Cu_2O	0.05	(15, 16)
CuO/Cu	0.02 – 0.16	(8, 9, 17)
NiO/Ni	0.02 – 0.16	(8, 9, 18)
Co_3O_4/CoO	0.01 – 0.02	(15, 19)
CoO/Co	0.16 – 0.17	(20, 21)
$Cu_2AlMnO_{5+\delta}$	0.024	(22)
$MgMnO_{2.68}/Mg_{0.5}Mn_{0.5}O$	0.0874	(23)
$CoTiO_{3}/Co^{+}TiO_2$	0.102	(24)
$Cu_{1.5}Mn_{1.5}O_4/CuMnO_2$	0.04	(25)
$(Mn_{0.77}Fe_{0.23})_3O_{4}/Mn_{0.77}Fe_{0.23}O$	0.067	(26)
$CaMn_{0.775}Ti_{0.125}Mg_{0.1}O_{2.9}$	0.096, 0.097, 0.085, 0.092, 0.085	(27)
$LaNiO_3$	0.083	(28)
$LaCu_{0.1}Ni_{0.9}O_3$	0.11	(28)
$LaCu_{0.5}Ni_{0.5}O_3$	0.10	(28)
$La_{0.1}Ca_{0.9}Cu_{0.1}Ni_{0.9}O_3$	0.0777	(29)
$SrFeO_3$	0.0294 and 0.0422	(30)
$SrFe_{0.9}Cu_{0.1}O_3$	0.0239, 0.0281, 0.038	(30)
$SrFe_{0.8}Cu_{0.2}O_3$	0.0270	(30)
$SrFe_{0.7}Cu_{0.3}O_3$	0.0393	(30)
$SrFe_{0.67}Cu_{0.33}O_3$	0.0401, 0.0486, 0.0486	(30)
$SrMnO_3$	0.0344, 0.0372, 0.0487	(31)
$SrNiO_3$	0.0823, 0.0829, 0.0847	(31)
$LaFeO_3$	0.059	(32)
$CaMn_{0.9}Mg_{0.1}O_3$	0.101 and 0.075	(33)
$CaTi_{0.125}Mn_{0.775}Mg_{0.1}O_3$	0.08	(34)
$Ca_{0.99}Li_{0.01}MnO_3$	0.0847 and 0.0878	(35)

Table S1: Experimental oxygen transfer capacities. Experimentally determined OTCs reported in literature for various metal oxides (7–35).

Supporting Notes

Note S1: Active learning workflow.

The generative core of this active-learning-driven materials discovery workflow is the WAE architecture (1) (see Figure S2 and Figure 1). This choice is advantageous due to its ability to learn meaningful latent representations, enforce smooth latent manifolds, and simultaneously reconstruct compositional and functional characteristics of candidate materials. By integrating structural descriptors with property predictions into a single entity, the WAE is adept at capturing complex relationships between composition and function, essential for the efficient exploration of the design space.

The input to the WAE encompasses two seven-dimensional vectors with normalized fractional compositions of elements occupying the A- and B-sites in the ABO_3 perovskites. Moreover, it includes a chosen number of scalar descriptor representing the target properties. Although only a single measure is used in the present study, namely the OTC or OC probability, successful tests have been performed where the Bartel tolerance (2) and configurational entropy were also included. As a result of being fed a combination of compositional and property information, the model is able to learn joint representations, enhancing its predictive accuracy and interpretability. The resulting input vector is compressed by the encoder E_θ into an eight-dimensional latent space. The former is, more precisely, structured as a series of fully connected layers of decreasing size (80, 64, 48 neurons), which allows the model to effectively learn intricate nonlinear relationships. Each is, moreover, accompanied by LN (3) and ReLU activation functions, to ensure training stability and convergence. In addition, a 10 % dropout regularization is applied to the first hidden layer, further improving the generalization performance by preventing overfitting and enhancing the robustness.

The resulting latent representation is constrained to align with an isotropic Gaussian distribution ($\mathcal{N}(0, I)$) via the definition of the training objective of the WAE. Specifically, the latter combines MMD regularization ($\mathcal{L}_{\mathrm{MMD}}$) with a L_1 loss ($\mathcal{L}_{\mathrm{recon}}$), which is applied between the original and reconstructed vectors (1)

$$\mathcal{L}_{\mathrm{total}} = \mathcal{L}_{\mathrm{recon}} + \lambda \cdot \mathcal{L}_{\mathrm{MMD}}. \tag{S1}$$

This choice is validated by the fact that the latter is particularly suitable for compositional data due to its robustness against outliers and balanced sensitivity to all vector dimensions. Together, these losses not only encourage the autoencoder to learn a latent representation that encodes both structural and functional information but also favor a continuous and smoothly varying latent manifold, beneficial for sampling and interpolation tasks during active learning (AL). Thanks to $\lambda \in [0, 1]$ it is, moreover, possible to control the balance between the accuracy of the reconstructed compositions, produced by the decoder from latent embeddings, ($\mathcal{L}_{\mathrm{recon}}$) and the alignment of the predefined prior with the encoded latent distribution ($\mathcal{L}_{\mathrm{MMD}}$). It should also be emphasized that the central Wasserstein characteristic of the autoencoder are afforded by the aforementioned Gaussian prior and MMD penalty, which are essential for achieving effective generative sampling and uncertainty quantification.

A distinctive feature of the WAE architecture presented here is the dual-decoder design, where each specializes in reconstructing different aspects of the input. The property decoder (PD_θ) is responsible for predicting the scalars directly from the latent representation. Its larger fully connected layers (192, 256, 320 neurons) are scaled by a factor of four compared to the composition decoder to provide sufficient representational capacity needed to capture complex, nonlinear relationships between latent space and physical properties. Conversely, the composition decoder (LD_θ) translates latent vectors back into physically meaningful fractional element compositions for both the A- and B-sites. It mirrors the encoder structure, maintaining consistency with layer sizes of 48, 64, and 80 neurons. The output from this decoder undergoes separate softmax normalization for each sublattice, enforcing compositional constraints and yielding valid and interpretable fractional compositions.

The implementation of the WAE within the AL pipeline significantly enhances materials discovery by facilitating effective exploration and interpolation within the compositional-property space. By providing smooth latent manifolds, it enables generative sampling of novel and physically plausible candidate materials, significantly improving predictive accuracy and interpretability. Additionally, the incorporation of dropout and LN (3) ensures stable training and robust generalization, critical in workflows constrained by the type of limited and noisy data that is typically encountered in materials discovery. In practice, the trained WAE model is integrated into the iterative active-learning loop, where it is coupled to Gaussian mixture models (GMMs) in the latent space, which is used for candidate generation. Specifically, this procedure also involves decoding the samples into compositions and properties that are, subsequently, filtered according to physical and chemical constraints before being validated by computationally demanding first-principles calculations. It should be emphasized that the iterative feedback thus achieved significantly improves data efficiency and accelerates the identification of promising materials.

The acquisition sampling represents an essential step in the AL cycle, as it decides which candidates are to be evaluated (36). Indeed, every iteration involves the generation n samples, each of which treated as a normally

distributed random variable. The mean (μ) and variance (σ) of the latter are, more precisely, provided by the surrogate model which predicts the properties of candidate materials and quantifies the predictive uncertainty. Based on extensive testing, Gaussian processes (GP) (37) has emerged as the method of choice since it has been found to yield more precise uncertainty estimates, which makes it better at pinpointing unexplored regions and, thus, guiding the AL cycle. Specifically, we utilized the Matérn-kernel based variant (37) that is implemented in GPyTorch (38) and features both an extra scaling parameter in addition to the individual length scales. An Adam optimizer (39) was, moreover, employed to select the hyperparameters, using negative marginal log likelihood as loss.

To ensure that the iterative refinement of the surrogate model is as efficient as possible the AL cycles, it is essential to use great care when querying new samples (40–42). Bayesian optimization (BO) represents a systematic method that achieves this objective by balancing exploration and exploitation; not only is it well suited for expensive-to-evaluate objective functions, it has also shown to be effective in guiding the discovery of materials in several experimental and computational studies (43–45). In essence, the idea is that the acquisition function picks points, from the model's predictive posterior, that are likely to either incur knowledge of the search space (exploration) or display optimal properties (exploitation). Thompson sampling is therefore an ideal choice, because it possesses all of the required properties but remains relative simple to both both understand and implement (46, 47). A key advantage is this method samples from the full posterior distribution of the surrogate model, instead of only relying on the predicted mean values (μ) or uncertainties (σ) of the selected points. This is achieved via a stochastic process during which the sampling is governed by the likelihood of providing an improvement, thus ensuring that both high-performing (exploitation) and uncertain (exploration) regions are explored. In technical terms, the normal distribution $\mathcal{N}(\mu, \sigma)$ provided by the trained surrogate model allows each of the candidates, which together make up a single sample, to be selected by first drawing n points and then picking the one with the highest drawn value:

$$\text{argmax}_x^{(n)}[f \sim \mathcal{N}(\mu, \sigma)]. \tag{S2}$$

Supporting References

[1] K. Muandet, K. Fukumizu, B. Sriperumbudur, and B. Schölkopf, *Kernel Mean Embedding of Distributions: A Review and Beyond,* Foundations and Trends in Machine Learning **10**, 1 (2017).

[2] C. J. Bartel, C. Sutton, B. R. Goldsmith, R. Ouyang, C. B. Musgrave, L. M. Ghiringhelli, and M. Scheffler, *New tolerance factor to predict the stability of perovskite oxides and halides,* Science Advances **5**, eaav0693 (2019).

[3] J. L. Ba, J. R. Kiros, and G. E. Hinton, *Layer Normalization,* 2016. `https://arxiv.org/abs/1607.06450`.

[4] B. Deng, P. Zhong, K. Jun, J. Riebesell, K. Han, C. J. Bartel, and G. Ceder, *CHGNet as a pretrained universal neural network potential for charge-informed atomistic modelling,* Nature Machine Intelligence **5**, 1031 (2023).

[5] S. P. Ong, W. D. Richards, A. Jain, G. Hautier, M. Kocher, S. Cholia, D. Gunter, V. L. Chevrier, K. A. Persson, and G. Ceder, *Python Materials Genomics (pymatgen): A robust, open-source python library for materials analysis,* Computational Materials Science **68**, 314 (2013). `doi:https://doi.org/10.1016/j.commatsci.2012.10.028`.

[6] A. Togo and I. Tanaka, *First principles phonon calculations in materials science,* Scripta Materialia **108**, 1 (2015).

[7] Z. Yu, Y. Yang, S. Yang, Q. Zhang, J. Zhao, Y. Fang, X. Hao, and G. Guan, *Iron-based oxygen carriers in chemical looping conversions: A review,* Carbon Resources Conversion **2**, 23 (2019). `doi:https://doi.org/10.1016/j.crcon.2018.11.004`.

[8] P. Cho, T. Mattisson, and A. Lyngfelt, *Comparison of iron-, nickel-, copper- and manganese-based oxygen carriers for chemical-looping combustion,* Fuel **83**, 1215 (2004). `doi:https://doi.org/10.1016/j.fuel.2003.11.013`.

[9] A. Abad, J. Adánez, F. García-Labiano, L. F. de Diego, P. Gayán, and J. Celaya, *Mapping of the range of operational conditions for Cu-, Fe-, and Ni-based oxygen carriers in chemical-looping combustion,* Chemical Engineering Science **62**, 533 (2007). Fluidized Bed Applications. `doi:https://doi.org/10.1016/j.ces.2006.09.019`.

[10] M. Johansson, T. Mattisson, and A. Lyngfelt, *Investigation of Fe_2O_3 with $MgAl_2O_4$ for Chemical-Looping Combustion,* Ind. Eng. Chem. Res. **43**, 6978 (2004). `doi:10.1021/ie049813c`.

[11] J. Adánez, L. F. de Diego, F. García-Labiano, P. Gayán, A. Abad, and J. M. Palacios, *Selection of Oxygen Carriers for Chemical-Looping Combustion,* Energy Fuels **18**, 371 (2004). `doi:10.1021/ef0301452`.

[12] E. R. Monazam, R. W. Breault, and R. Siriwardane, *Reduction of hematite (Fe_2O_3) to wüstite (FeO) by carbon monoxide (CO) for chemical looping combustion,* Chemical Engineering Journal **242**, 204 (2014). `doi:https://doi.org/10.1016/j.cej.2013.12.040`.

[13] T. Costa, P. Gayán, A. Abad, F. García-Labiano, L. de Diego, D. Melo, and J. Adánez, *Mn-based oxygen carriers prepared by impregnation for Chemical Looping Combustion with diverse fuels,* Fuel Processing Technology **178**, 236 (2018). `doi:https://doi.org/10.1016/j.fuproc.2018.05.019`.

[14] D. Mei, T. Mendiara, A. Abad, L. F. de Diego, F. García-Labiano, P. Gayán, J. Adánez, and H. Zhao, *Manganese Minerals as Oxygen Carriers for Chemical Looping Combustion of Coal,* Ind. Eng. Chem. Res. **55**, 6539 (2016). `doi:10.1021/acs.iecr.6b00263`.

[15] H. Song, K. Shah, E. Doroodchi, T. Wall, and B. Moghtaderi, *Reactivity of Al_2O_3- or SiO_2-Supported Cu-, Mn-, and Co-Based Oxygen Carriers for Chemical Looping Air Separation,* Energy Fuels **28**, 1284 (2014). `doi:10.1021/ef402268t`.

[16] X. Tian, M. Su, and H. Zhao, *Kinetics of redox reactions of $CuO@TiO_2Al_2O_3$ for chemical looping combustion and chemical looping with oxygen uncoupling,* Combustion and Flame **213**, 255 (2020). `doi:https://doi.org/10.1016/j.combustflame.2019.11.044`.

[17] Y. Zhang, E. Doroodchi, and B. Moghtaderi, *Chemical looping combustion of ultra low concentration of methane with Fe_2O_3/Al_2O_3 and CuO/SiO_2,* Applied Energy **113**, 1916 (2014). `doi:https://doi.org/10.1016/j.apenergy.2013.06.005`.

[18] M. M. Tijani, A. Aqsha, and N. Mahinpey, *Synthesis and study of metal-based oxygen carriers (Cu, Co, Fe, Ni) and their interaction with supported metal oxides (Al_2O_3, CeO_2, TiO_2, ZrO2) in a chemical looping combustion system,* Energy **138**, 873 (2017). `doi:https://doi.org/10.1016/j.energy.2017.07.100`.

[19] H. A. Alalwan, D. M. Cwiertny, and V. H. Grassian, *Co_3O_4 nanoparticles as oxygen carriers for chemical looping combustion: A materials characterization approach to understanding oxygen carrier performance,* Chemical Engineering Journal **319**, 279 (2017). `doi:https://doi.org/10.1016/j.cej.2017.02.134`.

[20] E. N. Son, S. H. Baek, R. Lee, J. I. Baek, H. J. Ryu, D. J. Yoo, and J. M. Sohn, *Study on the redox characteristics of CaCo based oxygen carrier for Chemical Looping Combustion,* Chemical Engineering Journal **377**, 121522 (2019). ISCRE 25 Special Issue: Bridging Science and Technology. `doi:https://doi.org/10.1016/j.cej.2019.04.102`.

[21] F. García-Labiano, L. F. de Diego, J. Adánez, A. Abad, and P. Gayán, *Temperature variations in the oxygen carrier particles during their reduction and oxidation in a chemical-looping combustion system,* Chemical Engineering Science **60**, 851 (2005). `doi:https://doi.org/10.1016/j.ces.2004.09.049`.

[22] S. Cloete, A. Giuffrida, M. Romano, P. Chiesa, M. Pishahang, and Y. Larring, *Integration of chemical looping oxygen production and chemical looping combustion in integrated gasification combined cycles,* Fuel **220**, 725 (2018). `doi:https://doi.org/10.1016/j.fuel.2018.02.048`.

[23] J. H. Hwang, J. I. Baek, H. J. Ryu, J. M. Sohn, and K.-T. Lee, *Development of $MgMnO_{3-\delta}$ as an oxygen carrier material for chemical looping combustion,* Fuel **231**, 290 (2018). `doi:https://doi.org/10.1016/j.fuel.2018.05.111`.

[24] J. H. Hwang, E. N. Son, R. Lee, S. H. Kim, J. I. Baek, H. J. Ryu, K. T. Lee, and J. M. Sohn, *A thermogravimetric study of $CoTiO_3$ as oxygen carrier for chemical looping combustion,* Catalysis Today **303**, 13 (2018). SI:The 16th Japan-Korea. `doi:https://doi.org/10.1016/j.cattod.2017.09.060`.

[25] I. naki Adánez-Rubio, A. Pérez-Astray, T. Mendiara, M. T. Izquierdo, A. Abad, P. Gayán, L. F. de Diego, F. García-Labiano, and J. Adánez, *Chemical looping combustion of biomass: CLOU experiments with a Cu-Mn mixed oxide,* Fuel Processing Technology **172**, 179 (2018). `doi:https://doi.org/10.1016/j.fuproc.2017.12.010`.

[26] R. Pérez-Vega, A. Abad, F. García-Labiano, P. Gayán, L. F. de Diego, M. T. Izquierdo, and J. Adánez, *Chemical Looping Combustion of gaseous and solid fuels with manganese-iron mixed oxide as oxygen carrier,* Energy Conversion and Management **159**, 221 (2018). `doi:https://doi.org/10.1016/j.enconman.2018.01.007`.

[27] A. Abad, A. Cabello, P. Gayán, F. García-Labiano, L. de Diego, T. Mendiara, and J. Adánez, *Kinetics of $CaMn_{0.775}Ti_{0.125}Mg_{0.1}O_{2.9-\delta}$ perovskite prepared at industrial scale and its implication on the performance of chemical looping combustion of methane,* Chemical Engineering Journal **394**, 124863 (2020). `doi:https://doi.org/10.1016/j.cej.2020.124863`.

[28] Q. Jiang, H. Zhang, Y. Deng, Q. Kang, H. Hong, and H. Jin, *Properties and reactivity of $LaCu_xNi_{1-x}O_3$ perovskites in chemical-looping combustion for mid-temperature solar-thermal energy storage,* Applied Energy **228**, 1506 (2018). `doi:https://doi.org/10.1016/j.apenergy.2018.07.028`.

[29] Q. Jiang, Y. Cao, X. Liu, H. Zhang, H. Hong, and H. Jin, *Chemical Looping Combustion over a Lanthanum Nickel Perovskite-Type Oxygen Carrier with Facilitated O_2-Transport,* Energy Fuels **34**, 8732 (2020). `doi:10.1021/acs.energyfuels.0c01038`.

[30] E. Ksepko, *Perovskite $Sr(Fe_{1-x}Cu_x)O_{3-\delta}$ materials for chemical looping combustion applications,* International Journal of Hydrogen Energy **43**, 9622 (2018). `doi:https://doi.org/10.1016/j.ijhydene.2018.04.046`.

[31] E. Ksepko, *Perovskite-type $Sr(Mn_{1-x}Ni_x)O_3$ materials and their chemical-looping oxygen transfer properties,* International Journal of Hydrogen Energy **39**, 8126 (2014). `doi:https://doi.org/10.1016/j.ijhydene.2014.03.093`.

[32] O. Mihai, D. Chen, and A. Holmen, *Chemical looping methane partial oxidation: The effect of the crystal size and O content of $LaFeO_3$,* Journal of Catalysis **293**, 175 (2012). doi:https://doi.org/10.1016/j.jcat.2012.06.022.

[33] L. F. de Diego, A. Abad, A. Cabello, P. Gayán, F. García-Labiano, and J. Adánez, *Reduction and Oxidation Kinetics of a $CaMn_{0.9}Mg_{0.1}O_{3-\delta}$ Oxygen Carrier for Chemical-Looping Combustion,* Ind. Eng. Chem. Res. **53**, 87 (2014). doi:10.1021/ie4015765.

[34] M. Keller, H. Leion, T. Mattisson, and H. Thunman, *Investigation of Natural and Synthetic Bed Materials for Their Utilization in Chemical Looping Reforming for Tar Elimination in Biomass-Derived Gasification Gas,* Energy Fuels **28**, 3833 (2014). doi:10.1021/ef500369c.

[35] B. S. Kwak, N.-K. Park, J.-I. Baek, H.-J. Ryu, and M. Kang, *Effect of oxidation states of Mn in $Ca_{1-x}Li_xMnO_3$ on chemical-looping combustion reactions,* Korean Journal of Chemical Engineering **34**, 1936 (2017). doi:10.1007/s11814-017-0107-0.

[36] B. Shahriari, K. Swersky, Z. Wang, R. P. Adams, and N. de Freitas, *Taking the Human Out of the Loop: A Review of Bayesian Optimization,* Proceedings of the IEEE **104**, 148 (2016). doi:10.1109/JPROC.2015.2494218.

[37] C. E. Rasmussen and C. K. I. Williams, *Gaussian Processes for Machine Learning* (The MIT Press, 2005). ISBN 9780262256834. doi:10.7551/mitpress/3206.001.0001.

[38] J. R. Gardner, G. Pleiss, D. Bindel, K. Q. Weinberger, and A. G. Wilson, *GPyTorch: blackbox matrix-matrix Gaussian process inference with GPU acceleration,* in *Proceedings of the 32nd International Conference on Neural Information Processing Systems*, NIPS'18, (Red Hook, NY, USA), 75877597, Curran Associates Inc., 2018.

[39] D. P. Kingma and J. Ba, *Adam: A Method for Stochastic Optimization,* 2017. https://arxiv.org/abs/1412.6980.

[40] P. I. Frazier, *A Tutorial on Bayesian Optimization,* 2018. https://arxiv.org/abs/1807.02811.

[41] B. Settles, *Automating Inquiry*, 1. Cham: Springer International Publishing, 2012. ISBN 978-3-031-01560-1. doi:10.1007/978-3-031-01560-1_1.

[42] F. Di Fiore, M. Nardelli, and L. Mainini, *Active Learning and Bayesian Optimization: A Unified Perspective to Learn with a Goal,* Archives of Computational Methods in Engineering **31**, 2985 (2024). doi:10.1007/s11831-024-10064-z.

[43] P. V. Balachandran, B. Kowalski, A. Sehirlioglu, and T. Lookman, *Experimental search for high-temperature ferroelectric perovskites guided by two-step machine learning,* Nature Communications **9**, 1668 (2018). doi:10.1038/s41467-018-03821-9.

[44] Y. Sverchkov and M. Craven, *A review of active learning approaches to experimental design for uncovering biological networks,* PLOS Computational Biology **13**, 1 (2017). doi:10.1371/journal.pcbi.1005466.

[45] Z. Rao, P.-Y. Tung, R. Xie, Y. Wei, H. Zhang, A. Ferrari, T. P. C. Klaver, F. Körmann, P. T. Sukumar, A. K. Silva, *et al.*, *Machine learning-enabled high-entropy alloy discovery,* Science **378**, 78 (2022).

[46] W. R. Thompson, *On the likelihood that one unknown probability exceeds another in view of the evidence of two samples,* Biometrika **25**, 285 (1933).

[47] A. Wang, H. Liang, A. McDannald, I. Takeuchi, and A. G. Kusne, *Benchmarking active learning strategies for materials optimization and discovery,* Oxford Open Materials Science **2**, itac006 (2022).
